# Heart repolarization changes after anthracycline therapy in the children with cancer

**Published:** 2014-07-20

**Authors:** H Amoozgar, S Zareifar, M Borzoie, F Qasem

**Affiliations:** 1 Division of Pediatric cardiology, department of Pediatrics, Shiraz University of Medical sciences, Shiraz, Iran.; 2 Hematology Research Center, Pediatric Hematology/ Oncology department, Shiraz University of Medical Sciences, Shiraz, Iran.; 3 Department of Pediatrics, Shiraz University of Medical Sciences, Shiraz, Iran

**Keywords:** Anthracyclines, QT dispersion, corrected QT dispersion, P dispersion

## Abstract

**Background:**

Anthracyclines are important components of many chemotherapeutic protocols. The present study aimed to evaluate the repolarization changes in electrocardiography (ECG) which may predict drug induced arrhythmia.

**Materials and Methods:**

In this cross-sectional study, the recorded ECGs were assessed for QT dispersion (QTd), QT corrected dispersion (QTcd), T peak to Tend dispersion (TPed), and P dispersion (Pd) in 12 ECG leads. The demographic information, including sex, age, and duration of drug consumption, were recorded, as well.

**Results:**

In this study, 112 patients, including 58 females (52%) and 54 males (48%) with the mean age of 8.7±4.5 years, as the case group were compared with 43 children, including 17 males (40%) and 26 females (60%), in the control group. Most of our patients (88%) had received usual doses of anthracyclines; i.e., 330 mg/m2. QT dispersion of the patients and the controls was 0.054±0.02 and 0.05± 0.02 seconds, respectively. No significant difference was found between the patients and the controls regarding corrected QT dispersion (P> 0.05). However, P dispersion time had increased in the patients' group. Our study showed that the duration of anthracyclines therapy did not cause any significant increase in ventricular re-polarization parameters.

**Conclusion:**

Anthracyclines may show their cardiac toxicity through increasing P dispersion.

## Introduction

Anthracyclines are commonly used antineoplastic drugs. However, their clinical utility is tempered by a dose-dependent risk of cardiotoxicity and congestive heart failure (1,2). Recent research has focused on early monitoring and risk stratification to identify patients that are 'at risk' for cardiotoxicity. Dose reduction, using biochemical markers or less cardiotoxic anthracycline analogues and prophylactic consumption of dexrazoxane are strategies which could be reduce anthracycline associated morbidity (3, 4).

Cardiac complications due to anthracyclines may develop several years after chemotherapy and are considered as a major cause morbidity and mortality in cancer patients after secondary malignancies and recurrence (5, 6). The mechanism of cardiac toxicity of these drugs has not been fully characterized (7,8). Some risk factors, such as age, dose, female gender, and radiation therapy, are known; however, the relative risks of many diseases, such as diabetes and hypertension, have not been well studied.

Mechanisms of anthracyclines on leukemia cells may intercalate into the DNA of replicating cells, leading to DNA fragmentation, inhibition of polymerases, and finally decrease in DNA, RNA, and protein synthesis (9, 10).

Although the exact mechanisms of anthracyclines damage to heart myocytes is unknown, there are several hypotheses (11). Elevations in serum cardiac troponins (T and I), representing a potential marker of damage, are associated with damage to heart cells (12). 

Anthracyclines cardio toxicities may occur in three stages: acute, sub-acute, and late (chronic). Acute and sub-acute toxicities may present with an abnormal electrocardiogram (ECG), ventricular and supra ventricular arrhythmias, ventricular dysfunction, and pericarditis- myocarditis syndrome (13-16). 

More than 30% decrease in QRS wave voltage may be an early evidence of cardiomyopathy in the patients receiving adriamycines (17, 18). The QT interval is a clinically important electrocardiographic measurement. In standard ECG, the QT interval shows the time between depolarization and end of repolarization. The usual method of ventricular repolarization measurement is based on the QT interval in lead 2 or in the lead which has the largest T wave (19). The QT should be corrected for the heart rate (QTc). Prolongation of the QTc (>440 ms) is equal to prolonged repolarization period of the ventricles which may result in fatal arrhythmias, including torsade de points, and a high mortality rate (20). 

QT dispersion is an indicator of general repolarization abnormality which may reflect the regional differences in ventricular recovery time and has been linked to the development of malignant arrhythmia in different cardiac diseases (21, 22). Depolarization abnormality may be reported sometimes in other condition such as children with syncope (23).

The normal range for QTd is 40-50 mili seconds and QTd of more than 65 milli seconds may increase the risk of arrhythmia and sudden death. 

The other criteria of repolarization are T peak to T end interval (TPE) and dispersion. TPE provides an index of maximum dispersion of repolarization and is an indicator of the transmural dispersion of the repolarization in different regions of the ventricular myocardium (24). 

Recent studies have used the P wave dispersion as a parameter for predicting atrial arrhythmias in several disorders (25-27).

The health related quality of life (QOL) has been accepted as an effective aspect of the patients' treatment, especially in chronic diseases, such as cancers (17). Cumulative dose of anthracyclines is a major risk factor for drug induced cardiac complications.

Thus, we performed this study in order to determine the repolarization changes after anthracyclines chemotherapy in children and probable practical use of these parameters for early diagnosis of anthracyclines cardiac toxicities.

## Materials and Methods

The present cross-sectional study was conducted one 112 patients less than 18 years old who were diagnosed as a case of childhood cancer and were under treatment with anthracyclines. All the patients with congenital heart diseases, history of radiotherapy, overt pulmonary or renal insufficiency, and congenital arrhythmia were excluded from the study. Written informed consent was obtained from the patients or their parents. In addition, the study was approved by the Ethics Committee of Shiraz University of Medical Sciences, Shiraz, Iran. 

The measured ECG parameters, including QT dispersion, P dispersion, QTc dispersion, and TPE dispersion, were compared between the case and control groups.

For all the study participants, their physical examination and medical history were recorded by a physician and a 12 lead digital standard ECG was obtained from the case and control groups. All the recordings were made at a paper speed of 25 mm/s with a digital electrocardiogram machine (Alicia Diagnostics, Sanford, FL, USA). The digitally recorded electrocardiogram tracings were evaluated using a digital clipper in Corel Photo Paint v.13 software (Ottawa, Canada). Magnification of the electrocardiogram made a fine determination of the measurement points. It should be noted that the electrocardiogram was interpreted by two cardiologists separately. 

Each measurement was repeated for 3 times and the mean values were calculated, as well. The QT interval was measured from the beginning of the QRs complex to the termination of the T wave (defined as the return to the isoelectric line). Bazett's formula was used to calculate the QTc: QTc= QT interval(s)/ (√R-R interval) (sec). The onset of the P wave was defined as the junction between the isoelectric line and the start of the P wave deflection. Besides, the offset of the P wave was defined as the point where the final deflection of the P wave crossed the isoelectric line. The leads with unclear onset or offset of the P wave were excluded from the study. Then, the P wave dispersion was calculated according to its definition as the difference between P maximum duration and P minimum duration in the 12 lead ECG. The T- peaked to T- end interval and T- peaked to T- end dispersion (defined as the difference between the maximum and minimum T- peaked to T- end interval in precordial leads V1 to V6 during a single beat ) were also measured. The T- peak to T–end was measured in each precordial lead and obtained from the difference between QT interval and QT peak interval, measured from the beginning of the QRS up to the peak of the T-wave.

A control group was selected from children with normal heart exam, no pathologic finding in echocardiography and normal ECG. 

The demographic data, including age and sex, and anthracyclines dose information, including <200 mg/m2, 200-300 mg/m2, and > 300 mg/m2, were recorded, as well. We also investigated the relationship between ECG findings and duration of anthracyclines treatment (less than one month, 1-6 months, and more than 6 months). The control group and these three groups of patients were matched for age.

The measured ECG parameters, including QT dispersion, P dispersion, QTc dispersion, and TPE dispersion, were compared in both study groups. 

**Statistical analysis**

Statistical analysis of the obtained data was performed under the supervision of a statistician using the SPSS statistical software (v. 15) (Chicago, IL, USA). ANOVA, T test, Pearson correlation, and linear regression were used in order to analyze the data. Besides, P value <0.05 was considered as statistically significant.

## Results

The current study was conducted on 112 patients, including 58 females (52%) and 54 males (48%) with the mean age of 8.7± 4.5years, who received anthracyclines for chemotherapy and 43 children, including 17 males (40%) and 26 females (60%) with the mean age of 8.6± 4.3, in the control group (p=0.8). 

Comparison of QT dispersion and QTc dispersion between the patients and the controls showed no statistically significant difference; however, P dispersion and TPE dispersion values were different between the two groups (Table I).

Moreover, the study results revealed an increase in P dispersion (0.02 second) and a decrease in TPE (0.09 second) in the drug receiving group.

**The effect of treatment duration:**

According to the duration of the treatment, the case group was divided into 3 groups including the 

patients with less than one month (31 cases), more than one month to 6 months (33 patients), and more than 6 months of treatment (48 cases).

According to the study findings, the QT dispersion in the patients who had received anthracyclines for less than a month was 0.048±0.017 which was significantly longer from the 1- to 6-month group (0.062±0.023) (P=0.025).

Furthermore, the P dispersion was higher in the 6- to 12-month group compared to the group which had not received any treatment (0.281±0.048 seconds versus 0.254±0.026, P=0.013). 

The results also showed that the TPE dispersion in the patients who had been treated for less than a month was 0.041 ± 0.061 which was significantly different from the control group (0.051±0.016).

The comparisons of the patients with less than one month, one to six months, and more than 6 months of treatment regarding the electrocardiographic parameters are presented in Table II. 

**Anthracyclines dosage:**

The three patient groups which had received less than 200 mg /m2 (n=63), 200 to 300 mg /m2 (n=30), and more than 300 mg /m2 (n=16) anthracycline and the control group were compared regarding the cumulative dose (mg /m2 of body surface area).

According to the results, P dispersion was prolonged by increasing the dosage beyond 200mg per square meter. 

The drug dosage did not have any significant effect on QT dispersion (p=0.42), QTc dispersion (p=0.59), and TPE dispersion (p=0.4).

**Table I:** Serologic Results test of anti-Toxoplasma Gondi IgM antibody in case and control groups

**Factor**	**Group**	**Mean(Second)**	**Std. Deviation**	**P **
**QT dispersion**	Patients	0.050	0.020	0.30
	Control	0.054	0.020	
**P dispersion**	Patients	0.254	0.026	**0.01**
	Control	0.276	0.042	
**QTc dispersion**	Patients	0.142	0.033	0.59
	Control	0.137	0.052	
**TPE dispersion**	Patients	0.052	0.016	**0.01**
	Control	0.043	0.016	

**Table II:** The electrocardiographic parameters patent with less than one month treatment, one to six month and more than 7 month treatment

**Factor**	**Category**	**Mean**	**Std. eviation**	**P**	**P **
**QT dispersion**	Less than 1 month	0.048	0.017	0.12	0.025 Difference was seen between less than one month and 1-6 month group (less than one month) < (1-6 month group)
	1-6 month	0.062	0.023		
	7- 12 month	0.053	0.019		
	No treatment	0.051	0.020		
**P dispersion**	Less than 1 month	0.272	0.049	0.23	0.013 Difference was seen between 6-12 month and no treatment (6-12 month) > (no treatment)
	1-6 month	0.273	0.040		
	7 - 12 month	0.281	0.048		
	No treatment	0.254	0.026		
**QTC dispersion**	Less than 1 month	0.129	0.050	0.99	0.437
	1-6 month	0.147	0.056		
	7 - 12 month	0.136	0.051		
	No treatment	0.142	0.033		
**TPE dispersion**	Less than 1 month	0.041	0.016	0.11	0.012 Difference was seen between less than one month and no treatment group (less than one month) < (no treatment group)
	1-6 month	0.042	0.017		
	6 - 12 month	0.045	0.014		
	No treatment	0.052	0.016		

## Discussion

This non-invasive study aimed to assess heart repolarization after anthracyclines treatment based on the measurement of QT dispersion, P dispersion, QTc dispersion, and TPE dispersion. First, the overall effect of these drugs on the heart was evaluated regardless of other factors, including age, sex, and drugs.

The study by Larsen and colleagues indicated that QTc prolongation, increased supraventricular, and ventricular tachycardia might occur in the patients on anthracyclines compared to the normal group (P<0.001). According to this study, no significant difference was found between the case and the control group regarding QT dispersion and QTc dispersion (p>0.05) (28).

In our study, the P dispersion of the drug treated group had increased compared to the control group (0.02 seconds). The researchers could find no studies about this variable after treatment with anthracyclines chemotherapy. A study by Senen and colleagues revealed that P dispersion was higher in the patients with dilated cardiomyopathy in comparison to the normal group (29).

The study results also showed that TPE dispersion had decreased in the drug receiving group (0.01 seconds). However, there are no researches evaluating the TPE dispersion after anthracyclines chemotherapy. A study showed that the TPE dispersion might increase in the patients with Brugada syndrome (30). TPE dispersion is a prediction factor for occurrence of arrhythmia. In our study, the significant TPE dispersion difference between the case and control groups was limited to less than 200 mg/m2 dosages. It should be mentioned that most of our patients (88%) had received less than the toxic dose of anthracyclines; i.e., 330 mg/m2 and only 12% of them received ≥ 330 mg/m2 anthracyclines. This may explain the cause of no increase in QT and QTc dispersion in most of our patients. 

Ko et al. conducted a study on the changes of QT dispersion after treatment with anthracyclines and showed that the QTc dispersion had a significant increase in the cases compared to the control group; however, this finding was not confirmed in Qt dispersion. In that study, the cumulative dose of anthracyclines was not involved in the increases of QTc dispersion and QT dispersion (31) which is in line with the results of the present study.

As shown in the current as well as the previous studies, repolarization factors are independent of gender 31. In the present study, the effect of treatment duration on ventricular repolarization factors was not statistically significant, which might be due to the short follow-up period. In fact, about 57% of our patients were evaluated for less than 6 months. In some studies, this effect was detected in more prolonged follow-up periods (28-30). 

Finally, our study findings showed no statistically significant correlation between the amount of drug as well as treatment duration and ECG parameters. 

 Conclusion: 

Anthracyclines can show their cardiotoxicity via an increase in P dispersion. The P dispersion is the earliest and consistent factor which is not affected by the gender of the patients, but has linear correlation with age.

## References

[1] Minotti G, Menna P, Salvatorelli E (2004). Anthracyclines: molecular advances and pharmacologic developments in antitumor activity and cardiotoxicity. Pharmacol Rev.

[2] Barry E, Alvarez JA, Scully RE (2007 Jun). Anthracycline-induced cardiotoxicity: course, pathophysiology, prevention and management. Expert Opin Pharmacother.

[3] Fulbright JM (2011). Review of cardiotoxicity in pediatric cancer patients: during and after therapy. Cardiol Res Pract.

[4] Levitt G (1999). Cardioprotection. Br J Haematol.

[5] Floyd JD, Nguyen DT, Lobins RL (2005). Cardiotoxicity of cancer therapy. J Clin Oncol.

[6] Monsuez JJ, Charniot JC, Vignat N (2010). Cardiac side- effects of cancer chemotherapy. Int J Cardiol.

[7] Singal PK, Iliskovic N (1998). Doxorubicin-induced cardiomyopathy. N Engl J Med.

[8] Puma N, Ruggiero A, Ridola V (2008). Anthracycline-related cardiotoxicity: risk factors and therapeutic options in childhood cancers. Signa Vitae.

[9] Lipshultz SE (2006 Jun). Exposure to anthracyclines during childhood causes cardiac injury. Semin Oncol.

[10] Hershman DL, McBride RB, Eisenberger A (2008). Doxorubicin, cardiac risk factors, and cardiac toxicity in elderly patients with diffuse B-cell non-Hodgkin's lymphoma. J Clin Oncol.

[11] Elliott P (2006 Jun). Pathogenesis of cardiotoxicity induced by anthracyclines. Semin Oncol.

[12] Cardinale D, Sandri MT, Martinoni A (2000). Left ventricular dysfunction predicted by early troponin I 13-release after high-dose chemotherapy. J Am Coll Cardiol.

[13] Nakamae H, Tsumura K, Terada Y (2005). Notable effects of angiotensin II receptor blocker, valsartan, on acute cardiotoxic changes after standard chemotherapy with cyclophosphamide, doxorubicin, vincristine, and prednisolone. Cancer.

[14] Guglin M, Aljayeh M, Saiyad S (2009). Introducing a new entity: chemotherapy-induced arrhythmia. Europace.

[15] Liu L, Liu ZZ, Liu YY (2013 Dec). Preventive effect of low-dose carvedilol combined with candesartan on the cardiotoxicity of anthracycline drugs in the adjuvant chemotherapy of breast cancer. Zhonghua Zhong Liu Za Zhi.

[16] Barrett-Lee P, Dixon J, Farrell C (2009). Expert opinion on the use of anthracyclines in patients with advanced breast cancer at cardiac risk. Ann Oncol.

[17] Carver JR, Shapiro CL, Ng A (2007). American Society of Clinical Oncology clinical evidence review on the ongoing care of adult cancer survivors: cardiac and pulmonary late effects. J Clin Oncol.

[18] Ali MK, Buzdar AU, Ewer MS (1983). Noninvasive cardiacevaluation of patients receiving adriamycin‐containing adjuvant chemotherapy (FAC) for stage II or III breast cancer. J Surg Oncol.

[19] Tristani-Firouzi M (2014 Apr 8). The long and short of It: Insights into the short QT Syndrome. J Am Coll Cardiol.

[20] Hage FG, de Mattos AM, Khamash H (2010 Jun). QT prolongation is an independent predictor of mortality in end-stage renal disease. Clin Cardiol.

[21] Malik M, Batchvarov VN (2000). Measurement, interpretation and clinical potential of QT dispersion. J Am Coll Cardiol.

[22] Monfared A, Atrkar Roshan Z, Salari A (2012 Apr). QT intervals in patients receiving a renal transplant. Exp Clin Transplant.

[23] Amoozgar H, Hosseiniasl M (2012 Sep). T-peak to T-end abnormality in pediatric patients with syncope. Iran J Pediatr.

[24] Watanabe N, Kobayashi Y, Tanno K (2004). Transmural dispersion of repolarization and ventricular tachyarrhythmias. J Electrocardiol.

[25] Ho TF, Chia EL, Yip WC (2001). Analysis of P wave and P dispersion in children with secundum atrial septal defect. Ann Noninvasive Electrocardiol.

[26] Andrikopoulos GK, Dilaveris PE, Richter DJ (2000). Increased variance of P wave duration on the electrocardiogram distinguishes patients with idiopathic paroxysmal atrial fibrillation. Pacing Clin Electrophysiol.

[27] Darbar D, Jahangir A, Hammill SC (2002). P wave signal-averaged electrocardiography to identify risk for atrial fibrillation. Pacing Clin Electrophysiol.

[28] Larsen RL, Jakacki RI, Vetter VL, Meadows AT, Silber JH, Barber G (1992). Electrocardiographic changes and arrhythmias after cancer therapy in children and young adults. Am J Cardiol.

[29] Senen K, Turhan H, Erbay AR (2004). P-wave duration and P-wave dispersion in patients with dilated cardiomyopathy. Eur J Heart Fail.

[30] Castro Hevia J, Antzelevitch C, Tornés Bárzaga F (2006). Tpeak-Tend and Tpeak-Tend dispersion as risk factors for ventricular tachycardia/ventricular fibrillation in patients with the Brugada syndrome. J Am Coll Cardiol.

[31] Ko JK, Kim YH, Park IS, Seo JJ, Moon HN (2001). QT Dispersion after Chemotherapy with Anthracyclines in Children. Journal of the Korean Pediatric Society.

